# Examination of the requirements for powered air-purifying respirator (PAPR) utilization as an alternative to lockdown

**DOI:** 10.1038/s41598-024-82348-0

**Published:** 2025-01-07

**Authors:** Yusaku Fujii

**Affiliations:** Gunma University, 1-5-1 Tenjin-Cho, Kiryu, 376-8515 Japan

**Keywords:** COVID-19, Lockdown, Powered Air-Purifying Respirator (PAPR), Infectious dose, Airborne transmission, Emergency evacuation, Herd immunity, Biological techniques, Biotechnology, Environmental sciences, Environmental social sciences, Diseases, Health care, Engineering

## Abstract

With the emergence of COVID-19 variants and new viruses, it remains uncertain when the next pandemic will occur. A lockdown is considered the last resort to halt the spread of infection; however, it causes significant economic and social damage. Therefore, exploring less harmful alternatives during such scenarios is crucial. This study explores the feasibility of using a powered air-purifying respirator (PAPR) as an alternative to lockdowns and as a strategy for infection control. Specifically, the study examines the potential impact of the PAPR wearing rate and PAPR aerosol shielding performance on the controllability of the spread of infections. The study investigated the necessary PAPR wearing rate and aerosol shielding performance to control infections as an alternative to the lockdown, using a simple simulation under limited conditions. When using a PAPR with 100% aerosol shielding capability in air supply, only 55% of the population needs to consistently wear the PAPR to reduce the effective reproduction number from a critical level (**R**_**t**_ = 2) to a target level (**R**_**t**_ = 0.9). Furthermore, if everyone consistently wears PAPR, only 55% the reduction ratio in the probability of becoming infected by PAPR supplying air **I**_**r_in**_ is enough for reducing the effective reproduction number from a critical level (**R**_**t**_ = 2) to a target level (**R**_**t**_ = 0.9). This study demonstrates the potential for utilizing PAPR as an alternative to lockdowns.

## Introduction

With the emergence of COVID-19 variants and new viruses, the occurrence of the next pandemic is uncertain, emphasizing the importance of preparedness for future outbreaks^1–3^.

Although it is challenging to quantitatively evaluate the effectiveness of lockdowns, which result in significant economic and social damage, they remain effective as a final measure to prevent the spread of infection^4^.

In this study, “lockdown” is defined as the imposition of strict restrictions on human and corporate activities to temporarily slow the spread of infection and allow time for other countermeasures, such as achieving held immunity through vaccination. A “lockdown” may include orders that prohibit individuals from leaving their homes and prevent corporations from operating. However, measures such as mask mandates and social distancing, which are part of the new lifestyle, are not considered as a part of a “lockdown”.

The nature and extent of lockdowns have been determined by governments on a case-by-case basis, depending on the severity of the outbreak and the balance between deterring infection and minimizing societal harm^5^. High fines have been reported to effectively enforce lockdown compliance among citizens^6^. The economic lockdown in the United States has shown both direct and indirect effects^7^.

Thus, finding less damaging alternatives to lockdowns is important to avoid the associated economic and social damage.

To reduce the effective reproduction number^8^, one of the following should be reduced:

**β** [/contact]: The probability of transmitting infection during contact with a susceptible individual.

**k** [contact/day]: The rate of contact with susceptible individuals.

**D** [day]: The duration of infectiousness.

Vaccination is expected to reduce **β** over the long term by enhancing immunity among susceptible individuals, while lockdowns are expected to temporarily reduce **k**.

This paper discusses a situation in which contact and oral infections are prevented through hand ishing and food hygiene, leaving airborne and droplet transmission as the primary routes of COVID-19 spread. The feasibility of using a powered air-purifying respirator (PAPR) as an alternative to lockdown is explored.

Powered air-purifying respirators (PAPR) are commercial devices designed to significantly reduce the number of viruses inhaled by the wearer, effectively preventing airborne transmission. They are widely used by medical personnel working at high-risk infection environments^9–11^.

Studies have reported nationwide support for the shortage of medical supplies for healthcare workers, including the PAPR, during the COVID-19 pandemic^12^. Some studies have highlighted the importance of establishing a system to increase production at the community level^13^. However, the use of PAPR as a lockdown alternative for the general public has not yet been considered.

Table 1 presents the specifications of a powered air-purifying respirator (PAPR) manufactured by 3 M^14^ and a low-cost PAPR developed by the author^15^ as examples of PAPRs.

**Table 1:**
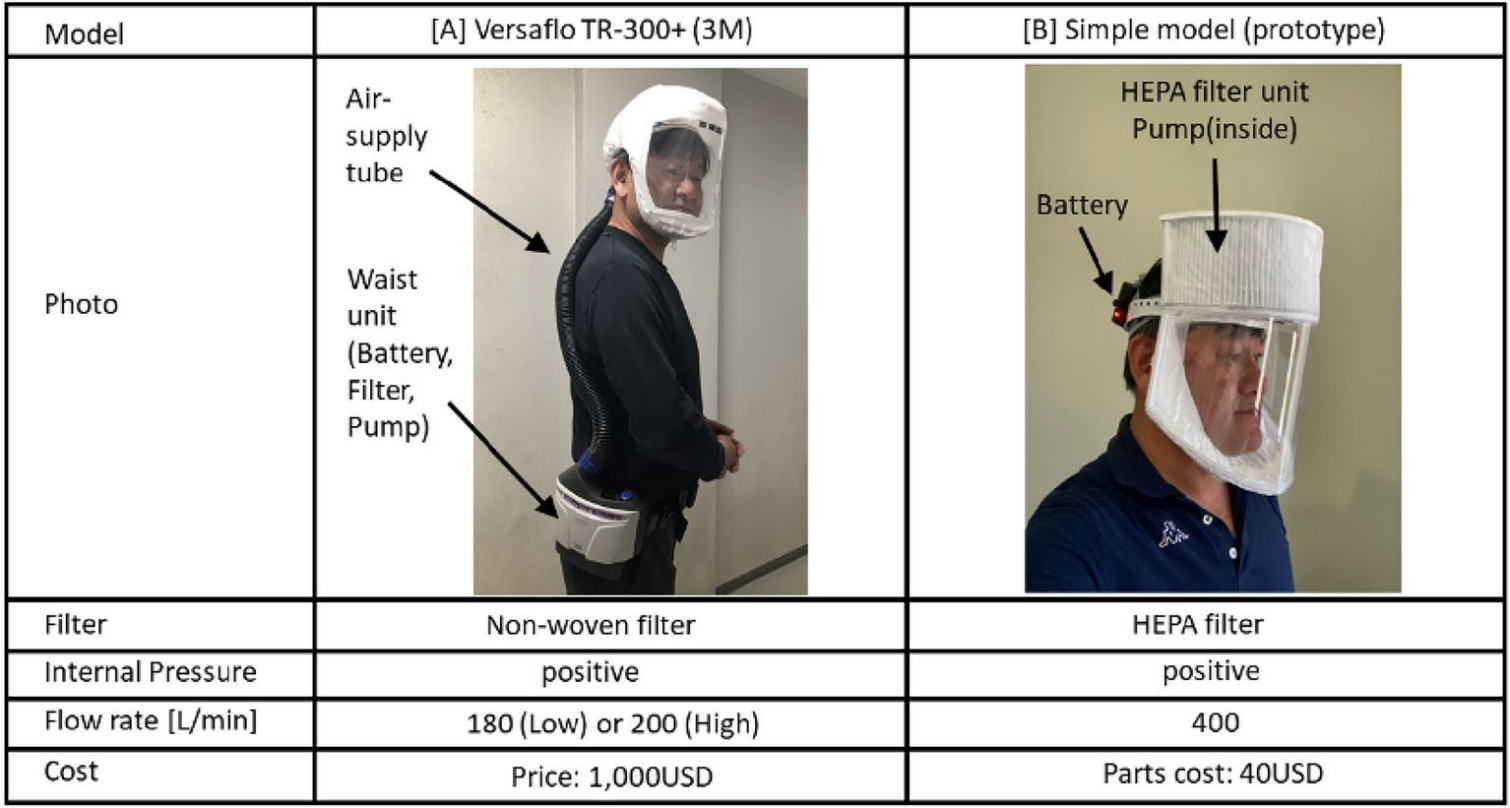
Specifications of two Powered Air-Purifying Respirators (PAPRs) (The PAPR wearer shown in the photos is Prof. Yusaku Fujii.)

The National Institute for Occupational Safety and Health (NIOSH) in the United States defines the Assigned Protection Factor (APF) as an index of the shielding performance of respiratory protective devices (PAPR, etc.)^16^. The APF is calculated as APF = [external concentration/internal concentration] for the target particulate matter (aerosol). For medical face masks, the APF = 10 when properly fitted by a trained wearer without gaps between the mask and the face. In contrast, a PAPR manufactured by 3 M has an APF of 1,000, demonstrating superior protection performance^14^. In other words, the concentration of aerosols containing viruses is reduced to ≤ 1/10 by a medical face mask (if worn with no gaps) and to ≤ 1/1000 by a PAPR. The high level of effectiveness is due to the PAPR hood being maintained at positive pressure, preventing outside air from directly entering through any gaps between the face and hood. The virus-shielding performance of PAPRs is considered consistent regardless of the type of virus, as they mechanically filter out particles containing viruses using a nonwoven fabric filter.

When assessing the aerosol shielding performance of nonwoven fabric filters, particles with a diameter of 0.3 μm—considered the most penetrating particle size (MPPS)—are generally targeted^17^. HEPA filters can filter out more than 99.97% of aerosols with a diameter of 0.3 μm.

The PAPR prototype developed by the authors^15^, as shown in Table 1, is a low-cost prototype model featuring a simple structure similar to that of commercially available medical PAPRs. It comprises a hood, a nonwoven fabric filter to supply purified air, a pump, and a battery. The total cost of the nonwoven filter, pump, battery, and other components is approximately 40 USD. The specifications of the air purification system are as follows^15^:

1. Only air that has been purified through a high-performance nonwoven filter (HEPA filter, capable of filtering more than 99.97% of aerosols with a diameter of 0.3 μm) is pumped into the hood.
2. Positive pressure is maintained inside the hood to prevent outside air from entering, even if there is a gap between the hood seal and the face of the wear.
3. Exhaust air is naturally exhausted through a thin nonwoven filter due to the positive pressure inside the hood, which helps deter the release of droplets or aerosols containing viruses, even if the wearer is infected.

The above features (1) and (2) are similar to those of the medical PAPR^14^ previously mentioned.

The proposed use of a high-performance PAPR as an alternative to the behavioral and activity restrictions of lockdowns for COVID-19 and other airborne diseases is based on the concept that such a device could provide effective infection control. The author investigated the feasibility of using PAPRs as an alternative to lockdowns and as a means of controlling airborne infections^18^. This study aimed to evaluate the required PAPR wearing rate, PAPR shielding performance, and other factors necessary to effectively control the spread of infection.

## Methods

A simple simulation model is developed under limited conditions to investigate the PAPR wearing rate and PAPR shielding performance, which are required to control infection as an alternative to lockdown. A social group in which contact and oral infections are completely prevented and airborne and droplet infections are the only routes of infection is used as the target. The factors affecting the effective reproduction number, other than the wearing of PAPR and the consequent non-use of face masks, such as the behavioral patterns of the social group in question, are assumed to remain unchanged.

Let **S**_**r_in**_ and **S**_**r_ex**_ denote the aerosol shielding rates in the supply/intake air and exhaust air of the PAPR, respectively.

**S**_**r_in**_: Shielding ratio of aerosols in the supply/intake air.

**S**_**r_ex**_: Shielding ratio of aerosol in the exhaust air.

The aerosol shielding ratio of the shielding rate of PAPR is normally considered for particles with a diameter of 0.3 μm, which is considered the most penetrating particle size (MPPS)^17^. A HEPA filter is capable of filtering > 99.97% of aerosols with a diameter of 0.3 μm, and the aerosol shielding ratio of a HEPA filter is considered to be 100%.

The "reduction in the probability of the wearer becoming infected by PAPR air supply compared to the condition before PAPR application, if the wearer is a susceptible person" and "reduction in the probability of infecting others by PAPR exhaust compared to the condition before PAPR application, if the wearer is an infected person" are defined as follows:

**I**_**r_in**_: The reduction rate in the probability that aerosols in the air supply infect a susceptible wearer compared with the pre-PAPR condition, such as wearing a face mask.

**I**_**r_ex**_: Reduction rate in the probability that an infected wearer infects others due to aerosols in the exhaust air compared to the pre-PAPR condition, such as wearing a face mask.

As for the wearing rate **W**_**r**_, if people in the population randomly wear the PAPR and the wearing rate is **W**_**r**_, this means that the same proportion of the susceptible population wears the PAPR and the same proportion of the infected population also wear the PAPR.

In actual operation, it is difficult to continue wearing the PAPR in situations, such as at home or while eating or drinking. Therefore, it is somewhat unreasonable to assume that the wearer must wear the PAPR at all times. However, by preparing a booth-type PAPR for individual use^19^ installed in each room of the home, including the bathroom, and eating or drinking places outside, it is possible to create conditions equivalent to the constant wearing of the PAPR by entering the booth-type PAPR at home or when eating or drinking.

Assuming that a proportion **W**_**r**_ of citizens wear PAPR with (**I**_**r_in**_, **I**_**r_ex**_) at all times, since the reduction in the probability of being infected by the air supply **I**_**r_in**_ applies to a proportion of susceptible persons, the effective reproduction number is modified as follows

1 $${\mathbf{R}}_{{{\mathbf{t}}\_{\mathbf{target}}}} = \, \left[ { \, \left( {{1} - {\mathbf{W}}_{{\mathbf{r}}} } \right) \, + \, \left( {{\mathbf{W}}_{{\mathbf{r}}} } \right)\left( {{1} - {\mathbf{I}}_{{{\mathbf{r}}\_{\mathbf{in}}}} } \right)} \right]{\mathbf{R}}_{{\mathbf{t}}} = \, \left[ {{1 } - \, \left( {{\mathbf{W}}_{{\mathbf{r}}} } \right)\left( {{\mathbf{I}}_{{{\mathbf{r}}\_{\mathbf{in}}}} } \right)} \right]{\mathbf{R}}_{{\mathbf{t}}}$$

Furthermore, because the reduction in the probability of infecting others by exhaust **I**_**r_ex**_ is applied to the same fraction **W**_**r**_ of infected persons, the effective reproduction number **R**_**t**_ is modified as follows

2 $${\mathbf{R}}_{{{\mathbf{t}}\_{\mathbf{target}}}} = \, \left[ {{1 } - \, \left( {{\mathbf{W}}_{{\mathbf{r}}} } \right)\left( {{\mathbf{I}}_{{{\mathbf{r}}\_{\mathbf{in}}}} } \right)} \right] \, \left[ {{1 } - \, \left( {{\mathbf{W}}_{{\mathbf{r}}} } \right)\left( {{\mathbf{I}}_{{{\mathbf{r}}\_{\mathbf{ex}}}} } \right)} \right]{\mathbf{R}}_{{\mathbf{t}}}$$

This equation is a quadratic equation for **W**_**r**_ and can be solved analytically if (**I**_**r_in**_, **I**_**r_ex**_) is known, by entering the current location and target value (**R**_**t**_, **R**_**t_target**_) of the number of reproductions performed.

However, determining (**I**_**r_in**_, **I**_**r_ex**_) is generally difficult. In the discussion section, a method for obtaining statistical data using the data collected from social experiments is presented.

The aerosol shielding rates **S**_**r_in**_ and **S**_**r_ex**_ in the supply and exhaust air can be measured. It is difficult to estimate the more important indices: **I**_**r_in**_, the reduction rate of the probability of being infected by aerosols in the supply air, and **I**_**r_ex**_, the reduction rate of the probability of infecting others by aerosols in the exhaust air.

However, the following limited arrangements can be made. First, assume a situation in which the face masks used in the population are replaced with PAPRs. Let **S**_**r_fm_in**_ and **S**_**r_fm_ex**_ denote the aerosol shielding ratios of the facemask to the air intake and exhaust, respectively.

For the air supply side, the following can be true.

1. If the aerosol shielding of the PAPR for air supply/intake is perfect (**S**_**r_in**_ = 1), the probability of being infected by aerosols in the intake air is reduced by 100% (**I**_**r_in**_ = 1) if a susceptible person wears PAPR.
2. If the aerosol shielding rate for the air supply/intake side of the PAPR **S**_**r_in**_ is the same as that of the facemask **S**_**r_fm_in**_ used in the population at that time (**S**_**r_in**_ = **S**_**r_fm_in**_), then the reduction in the probability of infection by aerosols in the air supply is zero (**I**_**r_in**_ = 0).
3. In the interval **S**_**r_fm_in**_ < **S**_**r_in**_ < 1 and, 0 < **I**_**r_in**_ < 1, a positive correlation exists between **S**_**r_in**_ and **I**_**r_in**_.

Similarly, for the air exhaust side, the following is true:

(4) If the aerosol shielding of the PAPR on air exhaust side is perfect (**S**_**r_ex**_ = 1), the probability of infecting others with aerosols in the exhaust is reduced by 100% (**I**_**r_ex**_ = 1) if the infected person wears the PAPR.
(5) If the exhaust-side aerosol shielding rate of the PAPR **S**_**r_ex**_ is the same as that of the facemask **S**_**r_fm_ex**_ used in the population at that time (**S**_**r_ex**_ = **S**_**r_fm_ex**_), then the reduction in the probability of infecting others with aerosols in the exhaust is zero (**I**_**r_in**_ = 0).
(6) In the interval **S**_**r_fm_ex**_ < **S**_**r_ex**_ < 1 and, 0 < **I**_**r_ex**_ < 1, a positive correlation will be observed between **S**_**r_ex**_ and **I**_**r_ex**_.

Using the above findings, simulations are performed in the next chapter.

## Results

Simulations are conducted under ideal conditions, such as a PAPR with 100% air supply shielding and a certain percentage of the population wearing the PAPR at all times. This simulation provides an outlook on the PAPR wearing rate and PAPR shielding performance required to sufficiently control the spread of infection.

### When a PAPR with 100% air supply shielding is always used by a certain percentage of the population

Assume that a certain proportion of the population in the social group **W**_**r**_ always wears a PAPR with (**S**_**r_in**_, **S**_**r_ex**_) = (1.0, **S**_**r_fm_ex**_), which results in.

1. A 100% reduction in the probability of infection due to air supply (**I**_**r_in**_ = 1) as a result of an aerosol shielding ratio **S**_**r_in**_ on the air supply side is 100% (**S**_**r_in**_ = 1).
2. The aerosol shielding ratio on the exhaust side of the PAPR** S**_**r_ex**_ is the same as that of the facemask used by the PAPR wearer before wearing it **S**_**r_fm_ex**_. This results in a 0% reduction in the probability of infecting others through exhaust (**I**_**r_ex**_ = 0).

Substituting (**I**_**r_in**_, **I**_**r_ex**_) = (1, 0) into Eq. (2) yields

3 $${\mathbf{R}}_{{{\mathbf{t}}\_{\mathbf{target}}}} = \, \left( {{1 } - {\mathbf{W}}_{{\mathbf{r}}} } \right){\mathbf{R}}_{{\mathbf{t}}}$$

The expression for **W**_**r**_ is as follows.

4 $${\mathbf{W}}_{{\mathbf{r}}} = { 1} - {\mathbf{R}}_{{{\mathbf{t}}\_{\mathbf{target}}}} /{\mathbf{R}}_{{\mathbf{t}}}$$

**Fig. 1:**
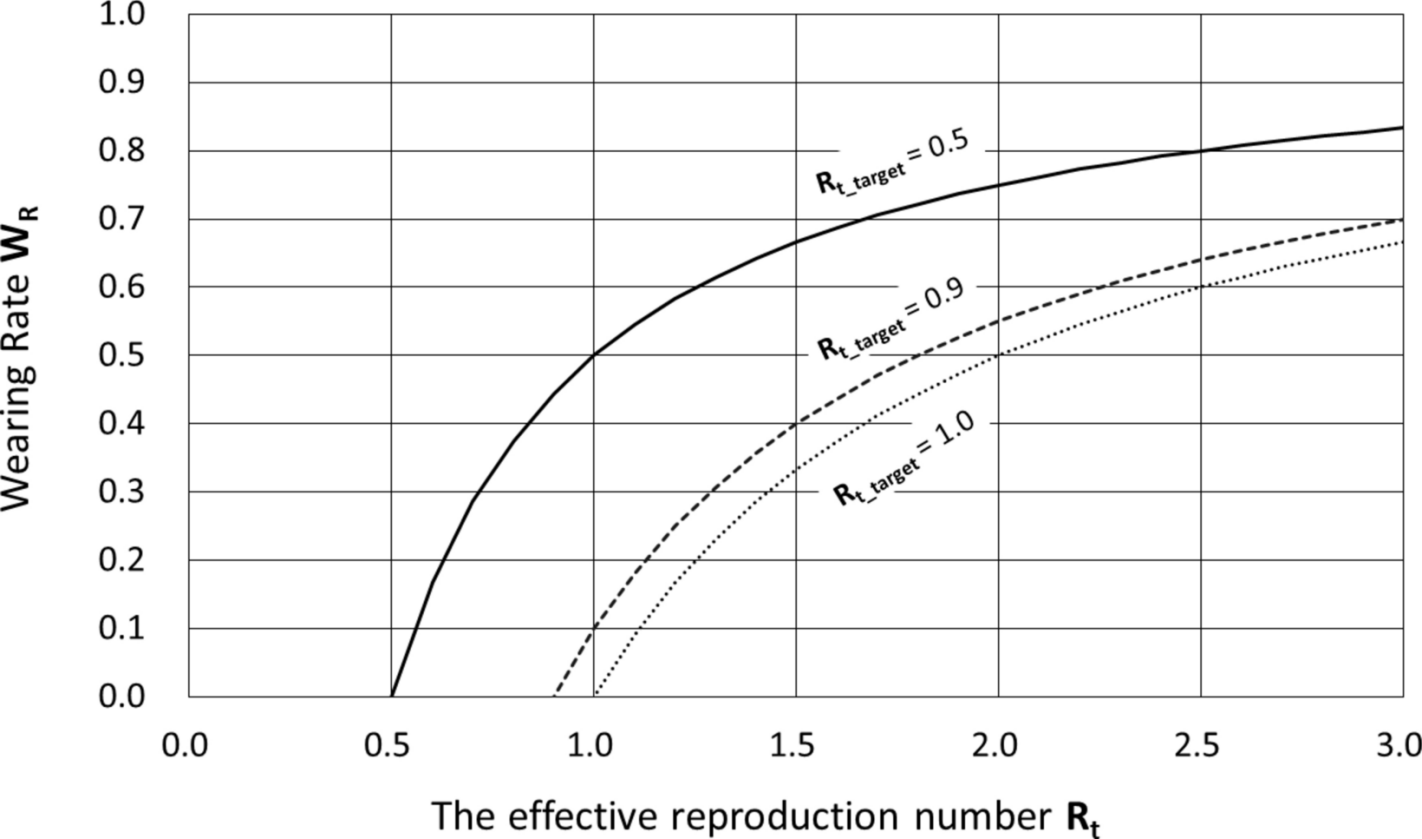
Wearing Rate **Wr** required for obtaining **Rt_target** = 0.5, 0.9 and 1.0 against **Rt**.

Figure 1 shows the relationship between **R**_**t**_ and **W**_**r**_ for **R**_**t_target**_ = 0.5, 0.9, and 1.0.

For example, if **R**_**t**_ = 2 at a certain point in time, 50%, 55%, and 75% of the population should wear the above PAPR at all times to achieve the target effective reproduction **R**_**t_target**_ = 1.0, 0.9, and 0.5, respectively. The effective reproduction number **R**_**t**_ = 2 is critical for the spread of the infection. It is noteworthy that only 55% of the population needs to wear PAPR at all times to achieve the target effective reproduction rate **R**_**t_target**_ = 0.9.

### When the PAPR of infection reduction rate (I_r_in_, I_r_ex_) is always used by a ratio W_r_ of citizens

Assuming that the proportion **W**_**r**_ of citizens always wears a PAPR with an infection reduction rate (**I**_**r_in**_, **I**_**r_ex**_), because the reduction in the probability of being infected by air supply **I**_**r_in**_ applies to the fraction **W**_**r**_ of susceptible persons, the effective reproduction number **R**_**t**_ is modified as follows

5 $${\mathbf{R}}_{{{\mathbf{t}}\_{\mathbf{target}}}} = \, \left[ { \, \left( {{1} - {\mathbf{W}}_{{\mathbf{r}}} } \right) \, + \, \left( {{\mathbf{W}}_{{\mathbf{r}}} } \right)\left( {{1} - {\mathbf{I}}_{{{\mathbf{r}}\_{\mathbf{in}}}} } \right)} \right]{\mathbf{R}}_{{\mathbf{t}}} = \, \left[ {{1 } - \, \left( {{\mathbf{W}}_{{\mathbf{r}}} } \right)\left( {{\mathbf{I}}_{{{\mathbf{r}}\_{\mathbf{in}}}} } \right)} \right]{\mathbf{R}}_{{\mathbf{t}}}$$

Furthermore, because the reduction in the probability of infecting others through exhaust **I**_**r_ex**_ is applied to the fraction **W**_**r**_ of infected persons, the effective reproduction number **R**_**t**_ is modified as follows

6 $${\mathbf{R}}_{{{\mathbf{t}}\_{\mathbf{target}}}} = \, \left[ {{1 } - \, \left( {{\mathbf{W}}_{{\mathbf{r}}} } \right)\left( {{\mathbf{I}}_{{{\mathbf{r}}\_{\mathbf{in}}}} } \right)} \right] \, \left[ {{1 } - \, \left( {{\mathbf{W}}_{{\mathbf{r}}} } \right)\left( {{\mathbf{I}}_{{{\mathbf{r}}\_{\mathbf{ex}}}} } \right)} \right]{\mathbf{R}}_{{\mathbf{t}}}$$

In particular, when there is no change in the probability of infecting others by exhaust, this is when **I**_**r_ex**_ = 0.0, we have

7 $${\mathbf{R}}_{{{\mathbf{t}}\_{\mathbf{target}}}} = \, \left[ {{1 } - \, \left( {{\mathbf{W}}_{{\mathbf{r}}} } \right)\left( {{\mathbf{I}}_{{{\mathbf{r}}\_{\mathbf{in}}}} } \right)} \right]{\mathbf{R}}_{{\mathbf{t}}}$$

8 $${\mathbf{W}}_{{\mathbf{r}}} = \, \left( {{1 } - {\mathbf{R}}_{{{\mathbf{t}}\_{\mathbf{target}}}} /{\mathbf{R}}_{{\mathbf{t}}} } \right) \, /{\mathbf{I}}_{{{\mathbf{r}}\_{\mathbf{in}}}}$$

**Fig. 2:**
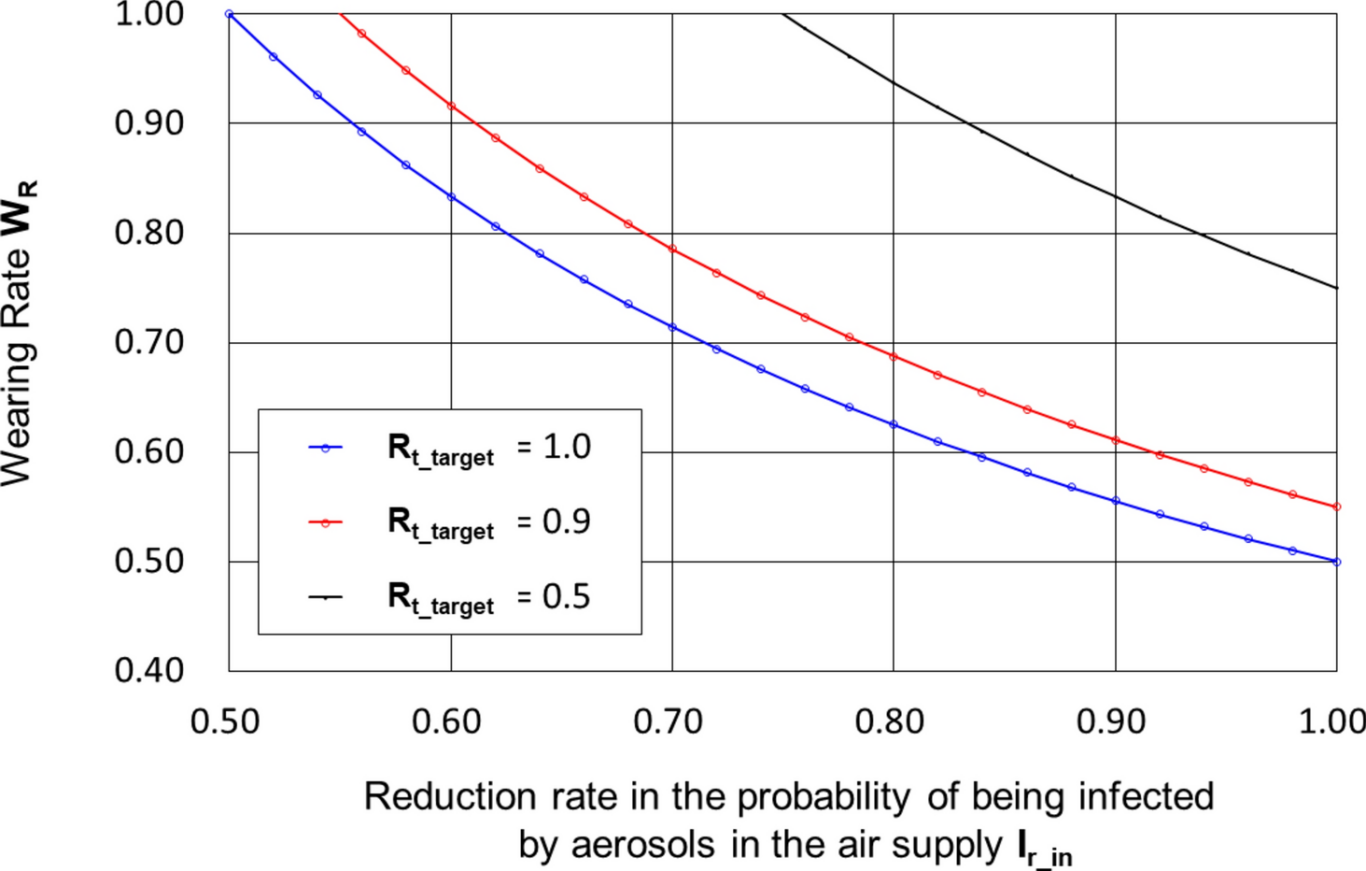
Wearing Rate **W**_**r**_ required for obtaining **R**_**t_target**_ = 0.5, 0.9 and 1.0 against **I**_**r_in**_.

Figure 2 shows the relationship between **I**_**r_in**_ and **W**_**r**_ for (**R**_**t**_, **I**_**r_in**_) = (2.0, 0.0) and** R**_**t_target**_ = 0.5, 0.9 and 1.0.

If everyone wears a PAPR, the reduction in the probability of infection due to PAPR air supply, **I**_**r_in**_, needed to adjust from a critical infection spread situation with an effective reproduction number **R**_**t**_ = 2 to a target effective reproduction rate **R**_**t_target**_ = 0.9, is only 55%.

However, it is difficult to determine the aerosol shielding ratio **S**_**r_in**_ on the air supply side to achieve **I**_**r_in**_ = 0.55. On the other hand, **I**_**r_in**_ ≥ 0.55 is considered easy to realize, because for a PAPR with an aerosol shielding ratio **S**_**r_in**_ of 100% (**S**_**r_in**_ = 1), **I**_**r_in**_ is considered to be 100% (**I**_**r_in**_ = 1).

## Discussions

### Empirical experiments to quantitatively evaluate the effectiveness of PAPR

**I**_**r_in**_ is the reduction rate in the probability of a susceptible wearer becoming infected by the PAPR air supply purification effect compared with that before the introduction of the PAPR, and **I**_**r_ex**_ is the reduction rate in the probability of an infected wearer infecting others by the PAPR exhaust air purification effect compared with that to before the introduction of the PAPR. (**I**_**r_in**_, **I**_**r_ex**_) are considered to be positively correlated with the aerosol shielding performance of PAPR air supply and exhaust (**S**_**r_in**_, **S**_**r_ex**_), and to be negatively correlated with the aerosol shielding performance of the face masks used in the social group before the introduction of PAPR when there is no change in the virus type causing the airborne infection or the social group’s behavior.

A method for estimating the reduction rate (**I**_**r_in**_, **I**_**r_ex**_) of infection probability for oneself and others due to air intake and exhaust of the PAPR using statistical data obtained from demonstration experiments and other sources is discussed. Here, it is assumed that contact and oral infection remain infection routes in addition to airborne and droplet infections.

In the previous formula (2), let **R**_**t_0**_ represent the effective reproduction number before correction with PAPR installation, and let **R**_**t_1**_ represent the effective reproduction number after correction with PAPR installation. It can be expressed as follows.

9 $${\mathbf{R}}_{{{\mathbf{t}}\_{\mathbf{1}}}} = \, \left[ {{1 }{-} \, \left( {{\mathbf{W}}_{{\mathbf{r}}} } \right)\left( {{\mathbf{I}}_{{{\mathbf{r}}\_{\mathbf{in}}}} } \right)} \right] \, \left[ {{1 }{-} \, \left( {{\mathbf{W}}_{{\mathbf{r}}} } \right)\left( {{\mathbf{I}}_{{{\mathbf{r}}\_{\mathbf{ex}}}} } \right)} \right]{\mathbf{R}}_{{{\mathbf{t}}\_{\mathbf{0}}}}$$

In formula (9), assume that in empirical experiments and similar studies, a dataset regarding the wearing rate **W**_**r**_, the effective reproduction number before correction with PAPR **R**_**t_0**_, and the effective reproduction number after correction **R**_**t_1**_ (**W**_**r**_, **R**_**t_0**_, **R**_**t_1**_) could be measured or estimated for each target population and for each analysis period. However, in the above formula, it is symmetric with respect to the 'reduction rate of infection probability for the wearer due to PAPR intake** I**_**r_in**_' and the 'reduction rate of infection probability for others around when the wearer is infected due to PAPR exhaust** I**_**r_ex**_' (**I**_**r_in**_, **I**_**r_ex**_), so it is not possible to determine (**I**_**r_in**_, **I**_**r_ex**_) using statistical methods such as the least squares method.

However, if the infection status of each individual in the social experiment group becomes clear and the above formula can be made asymmetrical with respect to the reduction rates of infection probability for the wearer and others due to PAPR intake and exhaust (**I**_**r_in**_, **I**_**r_ex**_), it is considered possible to determine (**I**_**r_in**_, **I**_**r_ex**_) using statistical methods.

For example, let us assume that the target population is **N** (persons) and that, for all citizens during the analysis period, susceptible and infected persons are identified, as well as whether they are wearing the PAPR. The number of susceptible persons is **N**_**1**_ (persons) and the number of infected persons is **N**_**2**_ (persons). Let **N**_**1_w**_ (person) and **N**_**2_w**_ (person) be the number of PAPR wearers among them, respectively, to obtain the PAPR wearer rates **W**_**r1**_ = **N**_**1_w**_/**N**_**1**_ for susceptible persons and **W**_**r2**_ = **N**_**2_w**_/**N**_**2**_ for infected persons.

The effect of reducing the effective reproduction number **R**_**t**_ by reducing the probability of susceptible persons becoming infected:

10 $${\mathbf{R}}_{{{\mathbf{t}}\_{\mathbf{1}}}} = {\mathbf{W}}_{{{\mathbf{r1}}}} \left( {{1} - {\mathbf{I}}_{{{\mathbf{r}}\_{\mathbf{in}}}} } \right){\mathbf{R}}_{{{\mathbf{t}}\_{\mathbf{0}}}} + \, \left( {{1} - {\mathbf{W}}_{{{\mathbf{r1}}}} } \right){\mathbf{R}}_{{{\mathbf{t}}\_{\mathbf{0}}}} = \, \left( {{1 } - {\mathbf{I}}_{{{\mathbf{r}}\_{\mathbf{in}}}} {\mathbf{W}}_{{{\mathbf{r1}}}} } \right){\mathbf{R}}_{{{\mathbf{t}}\_{\mathbf{0}}}}$$

Effect of reducing effective reproduction number **R**_**t**_ by reducing the probability of infected persons infecting others:

11 $${\mathbf{R}}_{{{\mathbf{t}}\_{\mathbf{1}}}} = {\mathbf{W}}_{{{\mathbf{r2}}}} \left( {{1} - {\mathbf{I}}_{{{\mathbf{r}}\_{\mathbf{ex}}}} } \right){\mathbf{R}}_{{{\mathbf{t}}\_{\mathbf{0}}}} + \, \left( {{1} - {\mathbf{W}}_{{{\mathbf{r2}}}} } \right){\mathbf{R}}_{{{\mathbf{t}}\_{\mathbf{0}}}} = \, \left( {{1 } - {\mathbf{I}}_{{{\mathbf{r}}\_{\mathbf{ex}}}} {\mathbf{W}}_{{{\mathbf{r2}}}} } \right){\mathbf{R}}_{{{\mathbf{t}}\_{\mathbf{0}}}}$$

Synthesizing the effects of **I**_**r_in**_ and **I**_**r_ex**_ leads to the following.

12 $${\mathbf{R}}_{{{\mathbf{t}}\_{\mathbf{1}}}} = \, \left( {{1 } - {\mathbf{I}}_{{{\mathbf{r}}\_{\mathbf{in}}}} {\mathbf{W}}_{{{\mathbf{r1}}}} } \right) \, \left( {{1 } - {\mathbf{I}}_{{{\mathbf{r}}\_{\mathbf{ex}}}} {\mathbf{W}}_{{{\mathbf{r2}}}} } \right){\mathbf{R}}_{{{\mathbf{t}}\_{\mathbf{0}}}}$$

In formula (12), assume that in empirical experiments and similar studies, a dataset concerning the PAPR wearing rate of susceptible individuals **W**_**r1**_, the PAPR wearing rate of infected individuals **W**_**r**2_, the effective reproduction number before PAPR installation **R**_**t_0**_, and the effective reproduction number corrected by PAPR installation **R**_**t_1**_ (**W**_**r1**_, **W**_**r2**_, **R**_**t_0**_, **R**_**t_1**_) could be measured for each target population and each analysis period. Since this formula is asymmetrical with respect to the reduction rates of infection probability for the wearer and others due to PAPR intake and exhaust (**I**_**r_in**_, **I**_**r_ex**_), it is possible to determine (**I**_**r_in**_, **I**_**r_ex**_) using statistical methods such as the least squares method.

Ideally, a dataset concerning the PAPR wearing rate of susceptible individuals **W**_**r1**_, the PAPR wearing rate of infected individuals **W**_**r2**_, the effective reproduction number before PAPR installation **R**_**t_0**_, and the effective reproduction number adjusted by PAPR installation **R**_**t_0**_ (**W**_**r1**_, **W**_**r2**_, **R**_**t_0**_, **R**_**t_1**_) should approximate a time series of instantaneous values. However, in practice, it is expected to be challenging due to the coarse time resolution of the available data and significant estimation errors. As procedures for a well-managed social experiment, the following can be suggested.

1. The target population of the experiment should be of a certain size and minimal interaction or movement with other populations. It is desirable to provide incentives to participants for their contributions and actions.
2. All residents underwent PCR testing at high frequency (daily) during the experimental period.
3. Based on (2), the effective reproduction number **R**_**t**_ is calculating, and its changes are recorded.
4. Provide PAPR free of charge to those who agree to wear it, and have them wear it all at once. The PAPR will be worn at all times during a short period (e.g., a few days) while they are out of the house.
5. Calculate **W**_**r1**_ and **W**_**r2**_ from the data obtained in (2) and (4), respectively.
6. Determine the effective reproduction number before simultaneous wearing **R**_**t_0**_ and the effective reproduction number after simultaneous wearing **R**_**t_1**_ from the procedure (2) above.
7. Conducting multiple sets of procedures (1)-(6): A certain period of PAPR non-wearing should be established between sets.
8. Using the obtained data sets (**W**_**r1**_, **W**_**r2**_, **R**_**t_0**_, **R**_**t_1**_) and Eq. (12), obtain (**I**_**r_in**_, **I**_**r_ex**_) using the least-squares method.

The social experiments described above should be conducted at various locations and times in conjunction with the free provision of PAPR and rewards for experiment collaborators, and the data sets (**W**_**r1**_, **W**_**r2**_, **R**_**t**_, **R**_**t_target**_) will be collected.

It is expected that a dataset consisting of 'reduction rate of infection probability for the wearer due to PAPR intake** I**_**r_in**_' and the 'reduction rate of infection probability for others around when the wearer is infected due to PAPR exhaust** I**_**r_ex**_' (**I**_**r_in**_, **I**_**r_ex**_) can be obtained according to the aerosol shielding performance of the PAPR air supply and exhaust (**S**_**r_in**_, **S**_**r_ex**_), and the public health status of the social group concerned, including the status of the airborne infection. Using the obtained (**I**_**r_in**_, **I**_**r_ex**_) as basic data, it is possible to optimize the specification and utilization of the PAPR according to the public health status of the social group in question.

The above discussion discusses how to quantitatively obtain the most important indices, (**I**_**r_in**_, **I**_**r_ex**_), through empirical experiments targeting the post-application to the pre-application of the PAPR. The same argument can be applied to the method of obtaining (**I**_**r_in**_, **I**_**r_ex**_) infection prevention measures other than the PAPR.

### Issues to be considered for acceptance by society

For infectious disease control measures to be accepted by society, it is essential not only to provide evidence of their high effectiveness in preventing infection and their low health risks, but also to consider factors such as the transparency and reliability of publicly available data, fairness across society, financial support by the government, flexibility to varying levels of infection spread, flexibility for individual differences (age, gender, physical condition, economic situation, etc.), sensitivity to cultural and social differences, and a balance between public safety, personal freedom, and privacy.

The existing PAPRs listed in Table 1 are too bulky and poorly designed for daily use by the general public, indicating significant potential for improvement. If PAPRs are recognized and accepted as an alternative to lockdowns, it is expected that their comfort, convenience, functionality, and design will improve rapidly and significantly, driven by the efforts of companies and governments. Some potential improvements include the following:

### Pressure control to aid breathing motion based on fluid models^18,20^

Consider the modeling of a PAPR with forced air supply through a non-woven filter by a pump and natural exhaust through a non-woven filter maintained by internal pressure (positive pressure). The following is a simple example of the modeling approach:

[A] Air supply flow rate **Q**_**in**_ (Δ**P**, **V**):

The flow rate through the filter is determined by the pressure difference Δ**P**_**f**_ before and after the filter. The flow rate through the pump is determined by the pressure difference Δ**P**_**p**_ before and after the pump as well as the applied voltage **V** to the pump. When the differential pressure Δ**P** (= Δ**P**_**f**_ + Δ**P**_**p**_) inside and outside the PAPR, along with pump’s applied voltage **V**, are determined, the air supply flow rate **Q**_**in**_ can be determined.

[B] Exhaust flow rate **Q**_**out**_ (Δ**P**):

The flow rate through the filter is determined by the pressure difference Δ**P**_**f**_ before and after the filter.

[C] Respiratory flow **Q**_**breath**_:

The flow difference **Q**_**diff**_ between the air-supply flow rate **Q**_**in**_ and the exhaust flow rate **Q**_**out**_ can be expressed as follows.

**Q**_**diff**_ = **Q**_**in**_ (Δ**P**, **V**)—**Q**_**out**_ = **Q**_**breath**_ + **Q**_**leak**_ + **Q**_**volume**_**.**

where,

**Q**_**breath**_: Respiratory flow rate of PAPR weares. Positive with inspiration.

**Q**_**leak**_: Leakage flow rate. Positive when leakage from inside to outside.

**Q**_**volume**_: Volume change inside PAPR. Positive with volume increase.

As the time averages of the respiratory flow **Q**_**breath**_ and volume change **Q**_**volume**_ are zero, the time average of **Q**_**diff**_ equals the time average of **Q**_**leak**_. Additionally, **Q**_**leak**_ can be expressed as a function **Q**_**leak**_ (Δ**P**) of differential pressure Δ**P**, assuming the shape of the gap between the face and mask is constant. Furthermore, the volume change **Q**_**volume**_ is considered a function **Q**_**volume**_ (Δ**P**), that is a function of the differential pressure Δ**P**. Thus,

**Q**_**breath**_ = **Q**_**diff**_—**Q**_**leak**_ (Δ**P**)—**Q**_**volume**_ (Δ**P**).

In this case, **Q**_**breath**_ < 0 indicates expiration, while **Q**_**breath**_ > 0 is indicates inspiration.

Thus, the exhalation and inhalation movements of the wearer can be detected in real-time. Based on this detection, the following controls can be implemented:

1. If exhalation is detected, a minimum positive pressure (e.g., 5 Pa) is set to minimize the resistance to the exhalation while preventing leakage from any gaps.
2. If inhalation is detected, a strong positive pressure setting (e.g., 50 Pa) is used to positively assist the inhalation process.

In addition to the forced air supply by the pump and filter, the introduction of a forced exhaust by the pump and filter enables the following differential pressure control:

1. When an exhalation is detected, a strong negative pressure setting (e.g. -50Pa) is used to actively assist the exhalation process.
2. When inhalation is detected, a strong positive pressure setting (e.g. 50Pa) is used to actively assist the inhalation process.

Using (1) and (2), a PAPR that facilitates both exhalation and inhalation of the wearer is realized. In this case, the direction of any potential leakage flow at gaps was opposite to normal, from outside to inside during exhalation, and from inside to outside during inhalation.

It is also possible to detect coughing from differential pressure Δ**P** measurements and estimate the possibility of infection in the wearer using other measurements, such as body temperature. The ability to efficiently identify infected persons will enhance targeted isolation and treatment, significantly reducing the spread of infection in the community.

Pressure control to aid breathing behavior based on the above fluid model is disclosed in a patent by the authors^20^. The English translation of the patented Claim-1 is “A system for recording and managing the wearing status of PAPR, characterized in that it determines the wearing status of PAPR, such as the shielding rate of particulates, droplets, viruses, etc., and whether or not PAPR is worn, based on the exhaust air flow rate and air supply air flow rate, and records them with the time on the recording device.”.

### Conversion to information terminal and introduction of the network management system^18,20^

The PAPR, especially the helmet-integrated PAPR shown in Table 1 [B], offers a power source and a fixed support structure on the head. This makes it feasible to integrate a smartphone or an augmented reality screen into the PAPR, effectively converting it into an information terminal. In addition to “providing clean breathing air,” the PAPR is anticipated to become an indispensable device in daily life as an information terminal.

A PAPR with a controller featuring Bluetooth connectivity and various sensors (such as differential pressure sensors, CO_2_ sensors, etc.) has already been developed^21^. A simple constant-pressure control algorithm has been implemented in the PAPR^21^. Future plans include incorporating the “reverse pressure control to assist breathing by fluid modeling” as described in the previous section. Furthermore, the Bluetooth connection function can enable connection to a wearing-rate network management system^18^ via the wearer’s smartphone. The Wearing Rate Network Management System (conceptual stage) aims to measure, analyze, and record the wear rate of each person and issues the electronic certification based on these data. This system is designed to balance individual freedoms—allowing people to decide when and where they choose not to wear the device—with the government’s need for effective infection control.

### Collaboration with other non-pharmaceutical interventions (NPIs)

It is important to collaborate on various Non-Pharmaceutical Interventions (NPIs) as countermeasures against infectious diseases, as well as medical countermeasures, such as vaccine-induced herd immunity or therapeutic drugs. Determining the appropriate combination of Non-Pharmaceutical Interventions (NPIs), such as social distancing, hand washing, disinfection, face masks, ventilation, quarantine, and PAPR, depending on the spread of infection is essential. The primary issue with face masks is the gap between the face and the mask through which the breathing air enters and exits the mask. However, face masks are effective in preventing infection, and they have the advantage of being easier to use than PAPR.

If the proposal in this paper is implemented, the government could offer the general public and businesses an alternative to lockdown using the event of a lockdown order. This would provide an option to minimize damage to society by allowing the government to offer the option of partially replacing each function of the lockdown with the use of PAPR, particularly in severe infection situations where lockdowns would typically be imposed.

This article discusses hood- and helmet-type PAPRs that cover the head. As mentioned previously, the author proposed a combination of both types^19^ that cover a desk/chair. The amount of purified air required is approximately proportional to the number of people (s) in the room multiplied by the amount of respiration per person. The concentration of carbon dioxide is as high as 50,000 ppm (5%) in exhaled air, compared with approximately 500 ppm (0.05%) in ambient air^22^. In helmet-type PAPRs that cover the head, the exhaled air with a high carbon dioxide concentration remains inside the helmet. To prevent an increase in the carbon dioxide concentration inside the helmet, a high flow rate (approximately 200–400 L/min) of air is supplied^22^ compared to the resting breathing rate (approximately 6–10 L/min)^14,15^. If the exhaled air could be smoothly discharged and prevented from diffusing into the helmet, the airflow rate could be reduced. A smaller airflow implies a smaller battery capacity, smaller pumps, and smaller filters of the PAPR, which contribute to the lower cost, smaller size, and lighter weight of the PAPR.

The components of booth-type PAPRs^19^ could be transferred to room ventilation units installed in ordinary houses and hotels. When pumps and filters are installed for both air supply and exhaust, the room can be set to a positive pressure setting when the inside room is to be protected and a negative pressure setting when the outside room is to be protected as a measure against air leakage through gaps, similar to pressure settings in a medical intensive care unit (ICU).

This paper explores the use of PAPRs as an alternative to the lockdown, mainly in public spaces. Regarding the use of PAPR in homes, the development of a room ventilation unit-type PAPR, as described, would make it possible to control virus concentrations in individual rooms. Air purification would also be expected to increase in enclosed spaces where many individual PAPRs (helmet-type PAPRs and booth-type PAPRs) are used, and where each PAPR purifies far more air than the amount exhaled by the user.

### To the world beyond

Although this discussion diverges from the main purpose of this study, it is worth noting that modern humans drink “purified water” rather than “natural water” directly from ponds and rivers. However, the “air” is not the same as "water," and modern humans breathe “natural air” just as primitive humans did.

If a highly comfortable and nearly perfect PAPR becomes commercially available, many people may prefer to wear it regardless of the pandemic. In other words, people might prefer to breathe purified air while drinking purified water. This could lead to a situation where many people are reluctant to inhale natural ambient air directly, just as they are reluctant to drink untreated water from natural rivers or ponds.

Humans have succeeded in greatly reducing the incidence of waterborne infectious diseases (cholera, dysentery, typhoid fever, etc.) as a result of drinking purified water. In the future, humans may succeed in drastically reducing the risk of airborne diseases by breathing purified air, and a society that is extremely resistant to airborne diseases may emerge.

## Conclusions

With the emergence of mutant strains of COVID-19 and new viruses, the timing of the next pandemic remains uncertain. Lockdowns, although an effective last resort to prevent the spread of infection, cause significant economic and social damage, making it crucial to identify less harmful alternatives.

This study examines the feasibility of using PAPRs as an alternative to lockdowns and as a Non-Pharmaceutical Intervention for infection control.

The required PAPR usage rate and its shielding performance for control infection as an alternative to the lockdown were investigated through simple simulations conducted under limited conditions, assuming that only airborne and droplet infections remained as infection routes.

For example, when using a PAPR with an intake shielding rate of 100%, it has been shown that a constant wearing rate of 55% is sufficient to adjust the effective reproduction number from a critical infection spread situation of **R**_**t**_ = 2 to the target effective reproduction number **R**_**t_target**_ = 0.9 In other words, if everyone wears a PAPR, an intake infection probability reduction rate **I**_**r_in**_ of 55% for the PAPR is sufficient to adjust the effective reproduction number from **R**_**t**_ = 2 in a critical infection spread situation to the target effective reproduction number **R**_**t_target**_ = 0.9.

This paper also discusses issues to be considered for public acceptance, collaboration with other Non-Pharmaceutical Interventions (NPIs), and the possibility that the widespread use of the PAPR will significantly reduce the risk of airborne infections in humans.

Additionally, the possibility of using PAPR as an alternative to lockdown and as a means of combating airborne infections was presented. In the future, it is expected that the feasibility of this approach will be demonstrated through social experiments, gain public understanding, and be endorsed by health organizations and the governments. It is anticipated that improvements to the PAPR devices by companies, the development and enhancement of network systems for monitoring wearing rates, and the establishment of corresponding social systems will progress, forming a resilient social infrastructure against airborne infectious diseases."

## Data Availability

The datasets used and/or analyzed during the current study available from the author on reasonable request.
